# Longitudinal relaxographic imaging of white matter hyperintensities in the elderly

**DOI:** 10.1186/2045-8118-11-24

**Published:** 2014-10-20

**Authors:** Valerie C Anderson, James T Obayashi, Jeffrey A Kaye, Joseph F Quinn, Phillip Berryhill, Louis P Riccelli, Dean Peterson, William D Rooney

**Affiliations:** 1 Advanced Imaging Research Center, L452, Oregon Health & Science University, 3181 SW Sam Jackson Park Rd, Portland, OR 97239, USA; 2 Department of Neurological Surgery, Oregon Health & Science University, Portland, OR, USA; 3 Department of Neurology, Oregon Health & Science University, Portland, OR, USA; 4 Portland VA Medical Center, Portland, OR, USA; 5 Department of Diagnostic Radiology, Oregon Health & Science University, Portland, OR, USA

**Keywords:** Aging, Blood–brain barrier, Blood volume, Relaxographic imaging, Periventricular, White matter hyperintensity, 7T

## Abstract

**Background:**

Incidental white matter hyperintensities (WMHs) are common findings on T_2_-weighted magnetic resonance images of the aged brain and have been associated with cognitive decline. While a variety of pathogenic mechanisms have been proposed, the origin of WMHs and the extent to which lesions in the deep and periventricular white matter reflect distinct etiologies remains unclear. Our aim was to quantify the fractional blood volume (v_b_) of small WMHs *in vivo* using a novel magnetic resonance imaging (MRI) approach and examine the contribution of blood–brain barrier disturbances to WMH formation in the deep and periventricular white matter.

**Methods:**

Twenty-three elderly volunteers (aged 59–82 years) underwent 7 Tesla relaxographic imaging and fluid-attenuated inversion recovery (FLAIR) MRI. Maps of longitudinal relaxation rate constant (R_1_) were prepared before contrast reagent (CR) injection and throughout CR washout. Voxelwise estimates of v_b_ were determined by fitting temporal changes in R_1_ values to a two-site model that incorporates the effects of transendothelial water exchange. Average v_b_ values in deep and periventricular WMHs were determined after semi-automated segmentation of FLAIR images. Ventricular permeability was estimated from the change in CSF R_1_ values during CR washout.

**Results:**

In the absence of CR, the total water fraction in both deep and periventricular WMHs was increased compared to normal appearing white matter (NAWM). The v_b_ of deep WMHs was 1.8 ± 0.6 mL/100 g and was significantly reduced compared to NAWM (2.4 ± 0.8 mL/100 g). In contrast, the v_b_ of periventricular WMHs was unchanged compared to NAWM, decreased with ventricular volume and showed a positive association with ventricular permeability.

**Conclusions:**

Hyperintensities in the deep WM appear to be driven by vascular compromise, while those in the periventricular WM are most likely the result of a compromised ependyma in which the small vessels remain relatively intact. These findings support varying contributions of blood–brain barrier and brain-CSF interface disturbances in the pathophysiology of deep and periventricular WMHs in the aged human brain.

## Background

White matter hyperintensities (WMHs) are commonly observed in the elderly brain on T_2_- weighted and proton density magnetic resonance imaging (MRI). The prevalence and severity of lesions increases with age and even mild increases in WMH volume may diminish cognition and global function in later life [1,2]. The presence of WMHs has been associated with a number of disease states in the elderly, including stroke and dementia. In patients with Alzheimer’s disease, WMH severity is often related to deficits in attention and frontal executive functions, leading to the suggestion that WMHs, in combination with amyloid deposition, may provide a ‘second hit’ to the degenerating brain, increasing the risk and/or clinical expression of the disease [3].

The etiology of incidental WMHs in the elderly is poorly understood. While neuropathological studies are informative, the myelin disruption, gliosis, and edema commonly observed in the parenchyma tend to reflect multiple inflammatory, neurodegenerative, and ischemic processes. In addition, the extent of these features varies with lesion severity, further obscuring the underlying mechanisms of their formation [4]. By and large, though, WMHs are a prominent feature of small vessel disease. Moreover, the severity of lesions is often associated with hypertension, diabetes, and hypercholesterolemia, providing additional support for the role of blood–brain barrier disturbances in their pathogenesis [5,6]. However, accumulating evidence suggests that the contribution of vascular factors to WMH formation may differ in the deep compared to the periventricular WM. In contrast to deep lesions (dWMHs), periventricular WMHs (pWMHs) are generally independent of infarct prevalence and plasma homocysteine levels, and show inconsistent association with hypertension and systolic blood pressure, especially in their early stages [7-11]. Moreover, histology regularly reveals defects in the adjacent ependymal lining suggesting that cerebrospinal fluid (CSF) infiltration may play a particularly important role in pWMH formation [12].

In recent years, quantitative MRI techniques have confirmed many of the histopathological features of WMHs *in vivo,* including myelin disruption (decreased magnetization transfer ratio) [13] and Wallerian degeneration (reduced fractional anisotropy) [14]. In addition, arterial spin labeling [15,16] and dynamic susceptibility contrast (DSC) MRI [17] have demonstrated WMH perfusion deficits compared to normal appearing white matter (NAWM). To extend these findings, we used T_1_ relaxographic imaging to measure the fractional blood volume (v_b_) of small, focal WMHs in the deep WM (centrum semiovale) and in mild hyperintense periventricular linings and caps. Relaxographic imaging is based on the measurement of the longitudinal relaxation rate constant of water protons (R_1_ ≡ 1/T_1_) before and after addition of a paramagnetic contrast reagent (CR). In cell suspensions and excised tissue, the technique provides information about intra- and extracellular volume and water residence times [18]. In combination with spatial encoding, the approach provides a quantitative measure of water exchange dynamics at the brain barriers. Relaxographic imaging has now been applied to a variety of human pathologies, including osteosarcoma, breast carcinoma, and multiple sclerosis [19-21].

Here, we measured R_1_ values throughout the brain before CR injection and during washout in a group of elderly adults with minimal cerebrovascular risk factors. Absolute values of v_b_ were then obtained by fitting the time-course of tissue R_1_ changes to a pharmacokinetic model that incorporates the effects of water exchange between the blood and extravascular compartments. Next, we segmented hyperintense from normal-appearing WM on fluid-attenuated inversion recovery (FLAIR) images and quantified the v_b_ in dWMHs and pWMHs on co-registered v_b_ maps. Finally, to investigate the potential role of degenerative factors and permeability changes at the ventricular CSF-tissue interface on pWMH formation, we examined the association of pWMH v_b_ with ventricular volume and with the rate of CR leakage into the CSF.

## Methods

### Participants

Twenty-three elderly individuals in good general health were enrolled. All participants underwent complete neurological and neuropsychological evaluation and provided a non-fasting blood sample for analysis of plasma homocysteine levels by fluorescence polarization immunoassay [22]. Subjects were excluded with body mass index greater than 30 kg/m^2^, uncontrolled hypertension, or history of diabetes mellitus, cardiovascular disease or TIA/stroke. Ten subjects were diagnosed with mild cognitive impairment (MCI) based on research criteria [23]. These individuals satisfied all criteria for study inclusion but had lower Mini-Mental State Examination (MMSE) [24] scores than cognitively normal subjects (26 ± 2 and 29 ± 1, respectively; *P* = .01). Three participants showed no radiographic evidence of WMHs, leaving a total of 20 datasets for analysis. Demographic features of these participants are shown in Table 1. The study was approved by the Oregon Health & Science University Institutional Review Board and all volunteers signed written informed consent.

**Table 1:** **Subject characteristics (N = 20)**^**a**^

**Characteristic**	
Female, male	8, 12
Age, y	71 ± 6
Education, y	17 ± 3
Hypertension, hyperlipidemia^b^	3, 5
MMSE	28 ± 2
Total homocysteine, μmol/L	10.8 ± 1.8
CSF volume, % ICV^c^	5 ± 3
WMH severity:^d^	
Deep	1 (0–2)
Periventricular	1 (0–2)

^a^Mean (SD) or median (range), unless otherwise noted; ^b^Number treated; ^c^ICV, intracranial volume; ^d^Fazekas visual rating scale.

### MRI data acquisition

All images were acquired using a 7 Tesla Magnetom scanner (Siemens Healthcare, Erlangen, Germany) with 8- or 24-channel transmit/receive head coil. High-resolution inversion recovery magnetization prepared rapid acquisition gradient echo (MPRAGE) [inversion time (TI) = 1050 ms, repetition time (TR) = 2300 ms, echo time (TE) = 2.8 ms, flip angle (FA) = 6°, field of view (FOV) = 256 × 256 mm^2^, reconstruction matrix = 320 × 320 pixels, and slice thickness = 0.8 mm] and FLAIR, [TE = 386 ms, TI = 2150 ms, TR = 8000 ms, FOV = 224 × 256 mm^2^, matrix = 280 × 320, slice thickness = 0.8 mm] images were used for volumetric measurements and WMH segmentation. The nominal spatial resolution of both sequences was 0.8 mm^3^. Dual T_2_- and proton density-weighted turbo spin echo with TE = 11 and 87 ms, TR = 10000 ms, FOV = 168 × 224 mm^2^, matrix = 256 × 192 mm^2^, slice thickness = 2 mm with 2 mm interslice gap were also obtained.

Variable inversion time MPRAGE datasets were acquired for T_1_-relaxography. Each dataset consisted of four axial MPRAGE images with TI = 300, 1800, or 3200 ms, or with no inversion pulse. Other sequence parameters were: TE = 2.3 ms, TR = 3500 ms, FA = 6°, FOV = 176 × 256 mm^2^, matrix = 176 × 256 pixels, slice thickness = 2 mm, resulting in a nominal spatial resolution of 1 × 1 × 2 mm^3^. All images were centered on the lateral ventricles, oriented parallel to the anterior commissure-posterior commissure line. MPRAGE datasets were acquired prior to a 0.11 mmol/kg dose of intravenous CR (gadoteridol; Bracco Diagnostics, Princeton, NJ, USA) and throughout the subsequent 50–60 minute washout period. The acquisition time for each dataset was 14.5 min.

### Image processing and analysis

**Figure 1:**
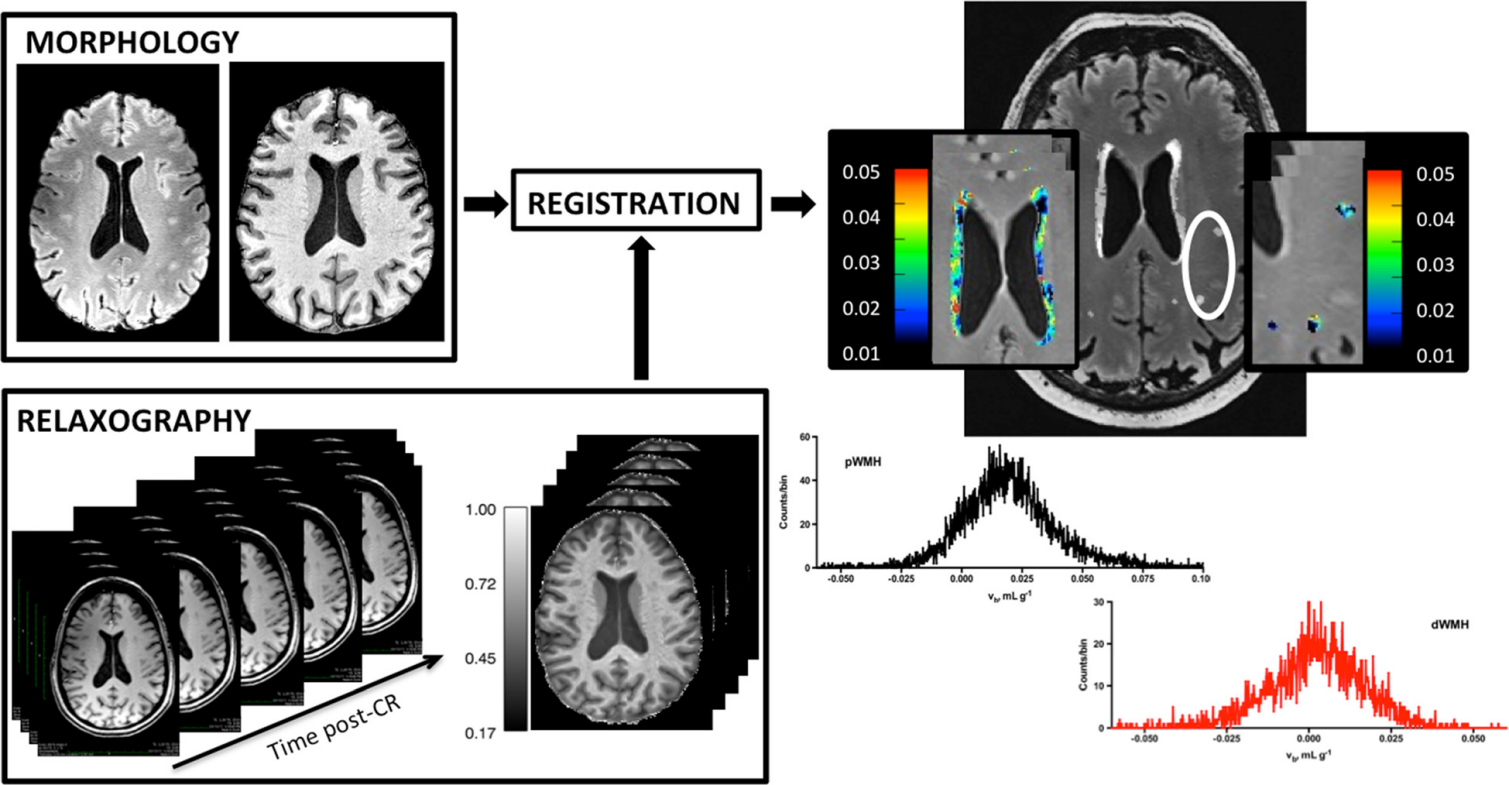
**Data workflow: (Left, top) FLAIR images were linearly registered to high-resolution MPRAGE space using rigid body registration.** (Left, lower) Co-registered R_1_ maps were prepared in high-resolution MPRAGE space after alignment of all variable-TI MPRAGE images to the TI 1800 image collected at the temporal mid-point of CR washout (see Methods for additional details). (Right) Fractional blood volume (v_b_) maps of pWMHs and dWMHs and corresponding FLAIR image. Insets show a magnified view of the WMHs (outlined in white) with associated color scales. The v_b_ histograms of each WMH are also shown.

An overview of the image analysis procedure is shown in Figure 1. Binary masks of NAWM and CSF were prepared using FMRIB’s Software Library (FSL; http://fsl.fmrib.ox.ac.uk/fsl/fslwiki/). Technical details of FSL image analysis tools have been described and validated previously [25]. Briefly, high-resolution MPRAGE images underwent bias correction [26], brain extraction (with eye and optic nerve cleanup) [27] and 3-class segmentation [28]. Use of default segmentation parameters resulted in exclusion of WMHs (which appear relatively hypointense on T_1_-weighted images) from the WM segment. NAWM masks were prepared as a subset of all WM voxels within 15 (±3) mm of the superior surface of the lateral ventricles. Ventricular CSF masks were prepared by manual removal of parenchymal pixels from the CSF binary image. CSF volume was calculated using partial volume estimates and normalized to the total intracranial volume. Binary WMH masks were prepared after linear registration (6 degrees of freedom) [29] of skull-stripped FLAIR images to high resolution MPRAGE space followed by segmentation of WMHs based on manual seeding and k-nearest-neighboring clustering (Seg3D; http://www.sci.utah.edu). Individual dWMH and pWMH masks were prepared by an experienced investigator. Careful attention was paid to avoid fluid-filled boundaries to minimize partial volume effects. Voxels that did not appear on at least three consecutive slices in all three orientations were not included. In 15 subjects, masks were independently prepared by a second trained investigator. Based on a 79 and 84% pixel overlap of dWMHs and pWMHs, respectively, we estimate segmentation reproducibility to be approximately 80%.

The severity of WMHs was assessed using the Fazekas rating scale [30]. WMHs were defined as WM areas with increased signal intensity on T_2_-spin echo and FLAIR images. Special care was taken to distinguish WMHs from lacunar infarcts, defined as parenchymal defects not extending to the cortical gray matter with a signal intensity following that of the CSF on all pulse sequences [31]. WMHs were present in all subjects; 15 participants had lesions in both deep and periventricular WM. In 17/20 subjects, lesions were seen as small scattered hyperintensities or as caps and pencil-thin linings around the ventricles (≤grade 1). In the remaining subjects, WMHs (3 periventricular, 3 deep) were more numerous and included a few patchy or more diffuse lesions. These were rated as grade 2. The total volume of pWMHs and dWMHs was 3.0 (±2.9) and 0.55 (±0.49) mL, respectively.

### Pharmacokinetic analysis

R_1_ maps were prepared from variable inversion time MPRAGE datasets. After brain extraction, all images in the dataset collected midway through CR washout (approximately 30 min post-CR) were linearly registered to the TI 1800 MPRAGE image. Each image in the remaining datasets was then registered to one of these images by application of the TI-dependent transform. R_1_ maps were prepared from each dataset by nonlinear least squares curve fitting (IDL; ITT Visual Information Solutions, Boulder CO, USA) of the signal intensity at each inversion time to the MPRAGE signal equation that includes magnetization losses due to sampling during recovery [32]. After preparation, all R_1_ maps were linearly registered to high-resolution MPRAGE space.

Full volume v_b_ maps were prepared by voxelwise fitting of water proton R_1_ values before and at each time after CR administration to a two-site (blood, extravascular) tissue model that accounts for the effects of transendothelial water exchange. The model assumes that each voxel exhibits monoexponential recovery after inversion and that CR remains confined to the intravascular space (i.e., K^trans^, the rate constant for CR extravasation, is negligible). Since tissue water molecules exchange across the capillary walls, the measured R_1_ value of tissue is a function of the relaxation behavior of water protons in each compartment. Due to the short range of the interactions responsible for longitudinal relaxation quenching by CR (typically on the order of several nanometers), only the R_1_ of blood is increased by an intravascular CR; the R_1_ of water protons in the extravascular space is unaffected. Therefore, the observed relaxation behavior of tissue becomes a function of the R_1_ value in the blood and the rate at which water exchanges across the capillary wall [33]. Under these conditions, it has been shown that the R_1_ behavior of water protons in tissue during CR washout from the blood, R_1t_(t), can be described by Eq. (1) where R_1b_(t) is the longitudinal relaxation rate constant of intravascular water at time t, R_1t_(0) and R_1b_(0) are the R_1_ values in tissue and blood before CR administration, and τ_b_^−1^ is the rate constant for water extravasation [33,34]. f_w_ is a scaling factor that accounts for the restricted localization of CR to the blood plasma. R_1e_ is the rate constant for extravascular water relaxation in the absence of exchange and is given by Eq. (2).

(1) $$\begin{array}{ll} R_{1t}\left(t\right) & =\frac{1}{2}\left\{\;\left[R_{1b}\left(t\right)+R_{1e}+\tau_{b}^{‒1}+\frac{v_{b}/f_{w}}{\tau_{b}\left(1‒v_{b}/f_{w}\right)}\right]\right)\\ & \;-\left[\;{\left(R_{1e}-R_{1b}\left(t\right)-\tau_{b}^{‒1}+\frac{v_{b}/f_{w}}{\tau_{b}\left(1‒v_{b}/f_{w}\right)}\right)}^{2}\right)\\ & \;\left({\left(+\frac{4v_{b}/f_{w}}{\tau_{b}^{2}\left(1‒v_{b}/f_{w}\right)}\right\}}^{\frac{1}{2}}\right] \end{array}$$

(2) $$\mathrm{where}\;R_{1e}=\left(\frac{R_{1t}\left(0\right)-R_{1b}\left(0\right)*\left(v_{b}/f_{w}\right)}{1-v_{b}/f_{w}}\right)$$

Values of v_b_ were iteratively determined using a gradient-expansion, non-linear least-squares algorithm [35] with τ_b_ and f_w_ held constant at reasonable values (0.3 and 0.8 s, respectively) [36,37]. R_1b_(t) values were measured from a region of interest contained entirely within the sagittal sinus. Uncertainties in R_1_ and v_b_ values were estimated using a Monte Carlo approach. The signal-to-noise ratio (SNR) of each MPRAGE image was calculated using the signal-background method [38]. Noise was then added by randomly sampling a Gaussian distribution with mean = 0 and standard deviation = signal intensity/SNR. Several voxels in each variable-TI dataset corresponding to WMHs were randomly selected and R_1t_ values estimated 1000 times. R_1t_ values were found to be robust to noise, with the variance in all voxels <0.04%. Errors in v_b_ were estimated by randomly selecting R_1_(t) values within 1 standard deviation of the mean and fitting to Eq. (1), as described above. Based on 1000 simulations, we estimate v_b_ variance to be approximately 1-2% of the mean.

### Ventricular permeability

Ventricular permeability was assessed based on region of interest (ROI) analysis of the temporal changes in CSF R_1_ values during CR washout. Unilateral ROIs (16 ± 9 μL) were defined in the posterior horns of the lateral ventricles on R_1_(t) maps. To minimize partial volume errors, ROIs were selected in areas that afforded optimal conspicuity of CSF-tissue interfaces. In the majority of cases, this was in the left posterior horn. Pixels with estimated fractional CSF volumes less than 1 were excluded to further reduce partial volume contamination.

### Statistical analyses

Data analysis was performed in Stata (StataCorp, College Station, TX, USA). Distributional outliers were identified by Mahalanobis and Cook’s Distance methods [39]. The mean v_b_ of WMH voxels was determined by fitting to a Gaussian distribution. Distributions that could not be normalized by common transformations (N = 3) were fit to non-negative, bounded (gamma or Weibull) distributions using EasyFit (Mathwave Technologies, Spokane, WA, USA). Voxels with nonphysiological v_b_ values (i.e., less than 0 or greater than 20 mL/100 g) were excluded from analysis. On average, these voxels accounted for 29 and 34% of all dWMH and pWMH voxels, respectively. Univariate associations were examined using linear regression analysis and the coefficient of determination, r^2^. Regional differences were examined using one-way within subject ANOVA with WM type (pWMH, dWMH, or NAWM) as the independent variable. *P* values < .05 were considered significant in statistical tests. Post-hoc pairwise comparisons were tested using paired t-tests. A modified Bonferroni procedure was used to adjust the level for (two-tailed) significance testing [40].

## Results

In the absence of CR, the average (±standard deviation) longitudinal relaxation time (T_1_ ≡ R_1_^−1^) of NAWM was 1454 (±54) ms. T_1_ values depend on field strength and vary somewhat with sequence and post-processing details. Nevertheless, this value corresponds well with the 1220 (±36) ms reported by Rooney and colleagues [41] and the 1500 (±100) ms reported by Li and Deoni [42]. Compared to NAWM, T_1_ times were increased in both pWMHs and dWMHs (1861 ± 124 ms and 1608 ± 80 ms, respectively; *P* < .0001). To our knowledge, T_1_ values of WMHs at 7Tesla have not been reported previously. Nevertheless, the 10-28% increase in WMH T_1_ values compared to NAWM measured here is consistent with the 5-40% increase in WMH T_1_ times noted at lower field strengths [43,44]. T_1_ values were significantly larger in pWMHs than dWMHs (*P* < .0001), although the between-subject variance was also increased in the former, most likely due to increased partial volume effects. Assuming a linear dependence of R_1_ values on tissue water fraction [45] and that T_1_ differences are due solely to alterations in water content, this corresponds to roughly a 15% increase in the total water fraction of pWMHs compared to dWMHs. Associations of WMH T_1_ values with age, gender, memory complaint (present or absent) and homocysteine levels were not significant.

**Figure 2:**
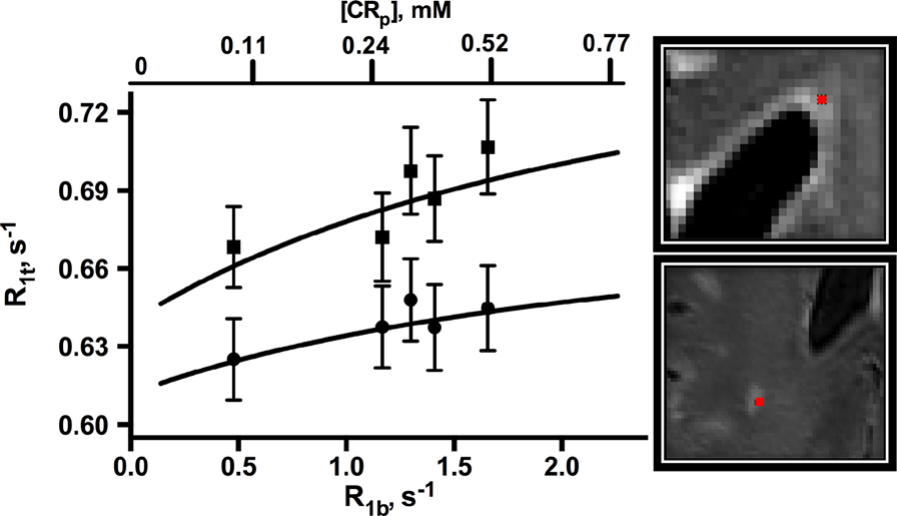
**Relationship between R**_**1t **_**and R**_**1b **_**values in a dWMH (circle) and pWMH (square) voxel (shown at far right).** Curves represent the best fit of the data in each WMH to Eq. (1). Error bars (±SD) are based on Monte Carlo simulations (see text). Upper axis represents plasma CR concentration, as calculated from Eq. (3).

As expected, R_1t_ values increased after CR administration in all subjects. Figure 2 shows the R_1t_ behavior in a representative dWMH and pWMH voxel plotted against the R_1b_ values. The best fit of the data in each voxel to Eq. (1) is also shown. The concentration of CR in plasma ([CR_p_]) depends on R_1b_[19] as:

(3) $$R_{1b}=\;R_{1b}\left(0\right)\;+\;r_{1}\left(1-h\right)\;\left[CR_{p}\right]$$

where h is the microvascular hematocrit (ca. 0.40) [46] and r_1_ is the relaxivity of CR at 7 T (3.3 s^−1^ mM^−1^ for gadoteridol) [47] and is plotted along the upper axis. The nonlinear relationship between R_1t_ and [CR_p_] observed here (especially prominent in pWMHs) is consistent with the compartmental nature of tissue and the restricted exchange of water between blood and extravascular sites.

The mean v_b_ values in NAWM averaged 2.4 (±0.6) mL/100 g, in good agreement with the 2.0 (±0.4) mL/100 g measured by DSC MRI [48] and the 2.7 (±0.4) mL/100 g found by two-point T_1_ relaxography in healthy adults [49]. In dWMHs and pWMHs, v_b_ averaged 1.8 (±0.6) and 2.4 (±0.8) mL/100 g, respectively. Mean v_b_ values varied significantly between dWMHs, pWMHs and NAWM (*F*_2,14_ = 6.33, *P* = .0054). Post-hoc tests showed a significant reduction in the v_b_ of dWMHs compared to NAWM (*P* < .0001). After correcting for multiple comparisons, differences between pWMHs and dWMHs were not significant (*P* = .033). Similarly, no significant associations of v_b_ with age, gender, or memory complaint were observed in periventricular or deep WMHs.

**Figure 3:**
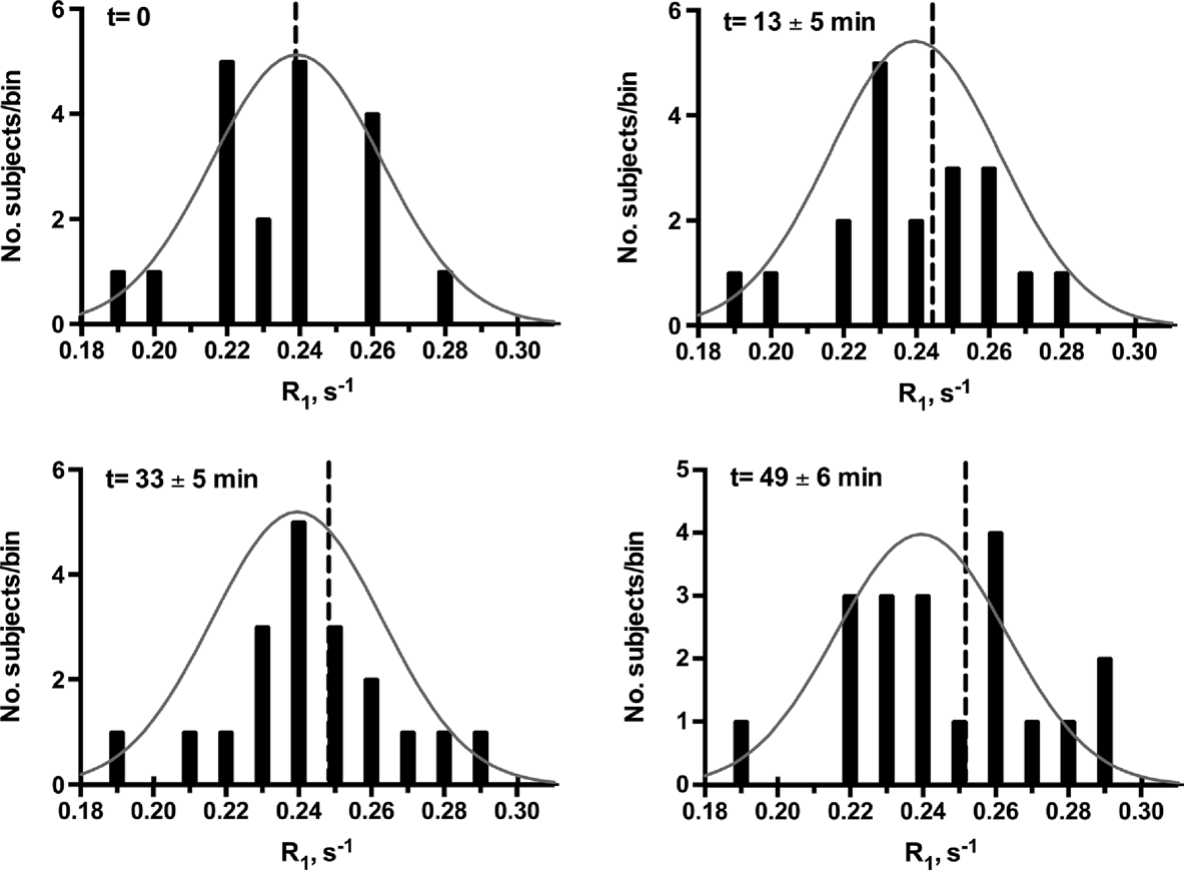
**Histograms of average CSF R**_**1 **_**values during CR washout.** The mean (dashed line) and fitted Gaussian distribution (solid line) are also shown. Time, t, was calculated as the temporal midpoint of each variable-TI image set. Data from one subject who completed only two post-CR measurements were excluded.

**Figure 4:**
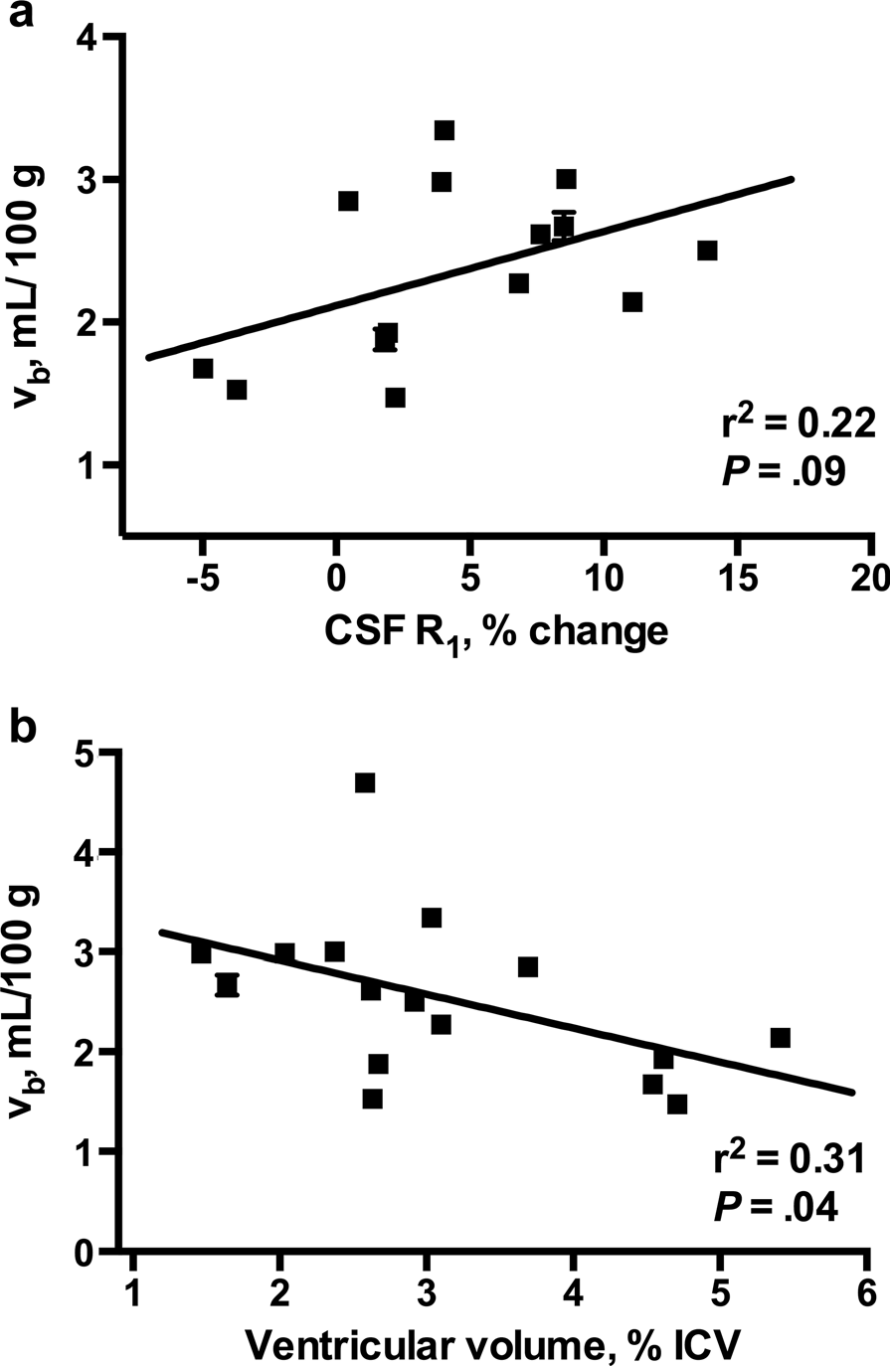
**Linear regression plots of pWMH v**_**b **_**and (a) percent change in CSF R**_**1 **_**values 33 (±4) minutes after CR administration, and (b) ventricular volume, as a percentage of the total intracranial volume (ICV).** Error bars represent the SEM.

In the absence of CR, CSF R_1_ values averaged 0.239 (±0.023) s^−1^, in good agreement with the 0.231- 0.237 s^−1^ reported previously [41,50]. These values increased to 0.244 (± 0.022) s^−1^ within 13 (±5) minutes after CR administration and continued to increase with time (*F*_*3*,18_ = 8.71, *P* < .0001**)**. Figure 3 shows the R_1_ histograms of ventricular CSF during CR washout. The total change in R_1_ values over the 50–55 minute measurement period was 0.0091 (±0.01) s^−1^. Assuming a linear dependence of CSF R_1_ values on CR concentration, this corresponds to a ventricular permeability of approximately 3.4 μM hr^−1^. Although not statistically significant (*P* = 0.09), pWMH v_b_ showed a moderate association (r^2^ = 0.22) with the R_1_ increase observed midway through the washout period (Figure 4a). An inverse association (r^2^ = 0.31; *P* = 0.04) of pWMH v_b_ with ventricular volume was also observed (Figure 4b).

## Discussion

Our results demonstrate that the fractional blood volume of small WMHs in the deep, but not periventricular WM, is decreased compared to NAWM in elderly individuals with minimal cardiovascular risk factors. While reduced v_b_ in the NAWM of individuals with and without WMHs has been demonstrated by ^15^O-positron emission tomography [51] and DSC MRI [48], the relative v_b_ of WMHs and NAWM has been examined in only a few studies. In migraine patients with larger (3–21 mm diameter) though still focal dWMHs, the v_b_ of lesions was found by DSC MRI to be reduced compared to NAWM, consistent with our results [44]. A similar finding has been reported in stroke subjects, although the diffuse nature of lesions in these subjects combined with the relatively poor spatial resolution makes comparisons more ambiguous [52]. However using the same technique in a group of healthy elderly individuals all between the ages of 85 and 86 years, Marstrand and colleagues were unable to find any significant reduction in the v_b_ of WMHs compared to NAWM [17]. Whether such differences are truly absent in the very elderly brain or were obscured by the corrections to their T_2_*-based acquisition parameters necessary to obtain absolute v_b_ measurements or the more limited spatial resolution of their images and subsequent partial volume effects, remains to be seen.

The absolute reduction in the v_b_ of dWMHs compared to pWMHs approached 25% in the present study. While confidence limits were too wide to establish the statistical significance of this finding (due at least in part because of small subject numbers), the magnitude of the difference suggests that alterations in blood–brain barrier permeability and/or decreased capillary density may be an important feature of lesion pathogenesis in the deep WM. In contrast, changes in capillary permeability appear to contribute relatively little to pWMH formation. Indeed, the microvascular defects and reduced capillary density frequently observed by histology in small dWMHs are generally not seen in early pWMHs [53]. Instead, hyperintense caps and pencil-thin periventricular linings are most commonly seen in association with a disrupted ependyma, presumably driven by abnormal osmotic pressure gradients or as a compensatory mechanism for the atrophic changes and ventricular enlargement that accompany normal aging [54]. Our findings of a significant inverse association between pWMH v_b_ and ventricular volume combined with the association of CSF [CR] and dilation of pWMH vessels are in accord with these findings. It is also possible that age-related or ischemic changes in the blood-CSF barrier may contribute to formation of pWMHs. Changes in the blood-CSF barrier are known to occur with age and cerebrovascular compromise and could explain the increased R_1_ values in the CSF [55]. However, since paracellular diffusion of relatively large molecular weight molecules (gadoteridol MW >500 Da) is likely to be minimal and transcellular mechanisms of CR transport across the blood CSF barrier unknown, additional experiments, ideally with increased temporal resolution in both the early and long times after CR administration, will be necessary to examine the role of the blood CSF barrier in pWMH formation more closely.

To our knowledge, the present study is the first to determine the absolute v_b_ of WMHs using relaxographic imaging. Our use of a T_1_- rather than T_2_*- based approach minimizes the systematic errors caused by bulk magnetic susceptibility effects which can degrade v_b_ estimates based on T_2_*-weighted images [56]. However, T_1_-based approaches typically suffer from low SNR and reduced detectability of CR. To increase the sensitivity of our measurements, all imaging was done at 7 T, where the SNR in WM is ca. 50% higher than at 4 T and the contrast to noise ratio is substantially increased [41,57]. The former results in improved detection of small lesions while the latter provides increased CR detection sensitivity and accuracy of R_1_ measurements. To further improve the accuracy of v_b_ estimates, R_1_ changes were fit to a pharmacokinetic model that incorporates the effects of transendothelial water exchange on the MR signal. Failure to account for exchange effects can result in large errors in v_b_ estimates, particularly in the early stages of lesion formation when vascular disturbances are expected to be small [58]. The model also assumes negligible leakage of CR from the vessels and a fixed rate of water exchange across the capillary walls. Such assumptions are reasonable in the healthy aged WM, where vascular changes are expected to be subtle. The extent to which they remain equally valid in both deep and pWMHs, though, remains unclear. Bolus tracking (dynamic contrast enhanced) MRI studies with increased temporal resolution are currently underway to examine the validity of these assumptions in both more closely.

Our results provide *in vivo* evidence that permeability changes in the blood–brain barrier are likely to accompany formation of dWMHs, while extravascular changes at the CSF-tissue interface may play a more important role in pWMH formation. However, our study is not without limitations. First, to minimize secondary effects of chronic ischemia, we excluded individuals with major cardiovascular risk factors. However, cognitive impairment was not an exclusion criterion and, although mild, was present in half of the study subjects. While the v_b_ of neither the NAWM nor WMHs was significantly different in these subjects, we did not measure cerebral blood flow. As a result, it is difficult to know to what extent our conclusions are affected by the hypoperfusion and altered cerebral autoregulation frequently associated with cognitive decline [59]. Second, the pharmacokinetic model upon which our estimates are based assumes that CR remains in the blood throughout the measurement period. If leakage into the extravascular space becomes significant, errors in v_b_ estimates can be substantial [36]. While CR extravasation across a relatively intact blood–brain barrier is likely to be minimal in the deep WM, a similar assumption in periventricular regions, where CR in the CSF may exchange with interstitial fluid at a disrupted ependymal lining, is less clear. As a result, the pathobiological basis of v_b_ associations in these regions should be interpreted with caution. Third, the validity of our findings depends on the accurate segmentation of WMHs. While the FLAIR images acquired at high magnetic field strength provide excellent conspicuity of small WM lesions, quantitative segmentation of WMHs remains challenging. Semiautomatic methods, like the one used here, rely on placement of a ‘seed point’ and propagation of the seeds to neighboring slices. The approach eliminates the need for manual outlining and reduces rater bias and error [60]. Nevertheless, results can be strongly dependent on the exact location of the seed point, as evidenced by an inter-rater agreement of selected pixels of only 80%. To reduce false positives, only those pixels identified as WMHs by both raters were included in our analyses. While this approach likely decreases the specificity of segmentation results, it provides an important increase in their sensitivity and the validity of WMH measurements. Fourth, the accuracy of R_1_ values determined from signal intensity changes in variable-TI datasets, and upon which v_b_ estimates rely, is made difficult by the complexity of the MPRAGE sequence [32]. To minimize these errors and improve the accuracy of R_1_ values, we used a fitting routine that models all the RF pulses, recovery periods and the approach to steady state magnetization assuming a constant flip angle across all imaging planes. However, we did not correct for RF inhomogeneities in the images which can be substantial at high field, particularly in central brain regions [57]. While the non-selective nature of the RF pulses and 3D image encoding make the MPRAGE sequence relatively insensitive to these inhomogeneities, they remain a potential source of R_1_ inaccuracies in our data. Finally, 3D relaxography, like all quantitative MRI, requires a balance between spatial and temporal resolution. To achieve the spatial resolution sufficient for identification of small WMHs, each variable-TI MPRAGE dataset required a scan times of almost 15 minutes. In agreement with measurements at 3 Tesla [61], the accuracy of the R_1_ values obtained by this method is quite good; however, the precision of v_b_ estimates would likely be increased with additional sampling frequency.

## Conclusions

Our findings suggest that in the absence of major vascular risk factors, the fractional blood water in WMHs is increased compared to NAWM in the healthy aged brain. While blood–brain barrier disturbances appear to be a prominent feature of WMHs in the deep WM, disturbances in the brain-CSF interface may play a more important role in the etiology of pWMHs.

## Abbreviations

CR: Contrast reagent; [CR_p_]: Contrast reagent concentration in plasma; CSF: Cerebrospinal fluid; dWMH: Deep white matter hyperintensity; DSC: Dynamic susceptibility contrast; FLAIR: Fluid-attenuated inversion recovery; ICV: Intracranial volume; MRI: Magnetic resonance imaging; MPRAGE: Magnetization prepared rapid acquisition gradient echo; NAWM: Normal appearing white matter; pWMH: Periventricular white matter hyperintensity; R_1b_: Blood longitudinal relaxation rate constant; R_1t_: Tissue longitudinal relaxation rate constant; ROI: Region of interest; SNR: Signal-to-noise ratio; T_1_: Longitudinal relaxation time; v_b_: Fractional blood volume.

## Competing interests

The authors declare that they have no competing interests.

## Authors’ contributions

VCA, JAK, JFQ and WDR designed the study. JTO developed the software for and prepared the R_1_ and v_b_ maps. LPR, PB, and DP assessed hyperintensity severity and performed lesion segmentations. VCA analyzed and interpreted the data, performed statistical analysis and drafted the manuscript. JAK, JFQ, and WDR revised the manuscript. All authors read and approved the final manuscript.

## Authors’ information

VCA- PhD (Chemistry), MCR (Clinical research)

JTO- BS

JAK- MD (Neurology)

JFQ- MD (Neurology)

PB- MD (Neurosurgery)

LPR- MD (Radiology)

DP- BA

WDR- PhD (Chemistry)
